# From writing paragraphs to evaluating articles: academic vocabulary development in EFL health sciences students during the ChatGPT transition

**DOI:** 10.3389/fmed.2026.1895483

**Published:** 2026-09-16

**Authors:** Khalid Azzubaidi

**Affiliations:** 1 King Abdullah International Medical Research Center, Riyadh, Saudi Arabia; 2 King Saud bin Abdulaziz University for Health Sciences, Riyadh, Saudi Arabia; 3 Ministry of National Guard–Health Affairs, Riyadh, Saudi Arabia

**Keywords:** academic word list, artificial intelligence, ChatGPT, EFL writing, English-medium instruction, health sciences education, vocabulary development

## Abstract

**Introduction:**

Evidence-based practice requires students to read, evaluate, and write about research, all of which depend on academic vocabulary. For English as a Foreign Language (EFL) health sciences students studying through English-medium instruction (EMI), this is a persistent challenge that affects their readiness for clinical practice, and AI writing tools have complicated it further since late 2022, as they can produce the academic English that writing courses spend years developing. This study tracks academic vocabulary development across four trimesters, a period coinciding with the phased rollout of ChatGPT in Saudi Arabia.

**Methods:**

The corpus-based analysis drew on 855,680 running words from the King Saud bin Abdulaziz University for Health Sciences Learner Corpus, produced by 157 female preparatory program students with Arabic as their first language. Academic Word List (AWL) coverage was computed for each trimester as the percentage of running words drawn from the 570 word families of the AWL, and compared against published benchmarks for medical academic texts. Each trimester was mapped to one of four phases of ChatGPT accessibility.

**Results:**

Overall AWL coverage was 7.66%, rising significantly from 3.69% in Trimester 1 to 11.39% in Trimester 4. The steepest gain coincided with the most demanding writing task and with unrestricted ChatGPT access, and Trimester 4 coverage exceeded the 10.07% benchmark for expert medical research articles.

**Discussion:**

Progressive, genre-based English for Academic Purposes (EAP) instruction was associated with substantial vocabulary growth. Because task complexity, cumulative language development, and AI availability all increased over the same period, their effects cannot be disentangled, and the results are interpreted as associations rather than as evidence of an AI effect. The findings carry implications for vocabulary teaching and for the validity of unsupervised writing assessment.

## Introduction

A health sciences student who cannot understand the word “significant” in a clinical trial report or use “participants” when writing up a study is not yet ready for evidence-based practice. Critical appraisal of medical literature is a core competency in undergraduate medical education (1, 2). It involves reading research methods sections, interpreting statistical findings, and writing coherent critiques. All of this depends on having a working command of academic vocabulary. Without it, students end up memorizing clinical guidelines instead of being able to evaluate the evidence behind them. The question for medical educators, then, is not whether academic vocabulary matters. It is whether their curricula are actually developing it, and how to tell.

This challenge is most acute in English-medium instruction (EMI) settings, where content is taught through English regardless of students’ first language (L1). Some students in EMI classrooms have English as their L1; others meet it as an additional language. Where English circulates widely outside the classroom, these learners are second language (L2) users; where it is met almost only in class, as in Saudi Arabia, they are learners of English as a foreign language (EFL). What separates the two is exposure rather than the process of learning, and in both cases disciplinary content and the academic English carrying it must be acquired at once. EMI adoption has expanded substantially across Saudi health sciences universities, though evidence of its effectiveness remains inconsistent (3, 4). Alkhateeb and Alhawsawi (3) showed just how steep the transition is from Arabic-medium high school to English-medium medical education. Gaffas (5) surveyed 264 Saudi EFL medical students and found writing to be among the most challenging skills in their EMI studies. Cognitive load theory helps explain why, since processing difficult medical content in a second language taxes working memory from two directions at once. A systematic review of a decade of EMI research in healthcare education found evidence that when academic English demands exceed students’ proficiency, motivation and confidence tend to decline (6). Klarare et al. (7), in a scoping review of academic literacies in health professions training, highlighted that the biggest gap for incoming students is their inability to read, interpret, and critically evaluate academic texts. Compounding this challenge, health sciences students face a double vocabulary burden: they must learn the technical language of medicine (anatomy, pharmacology, clinical classifications) while also developing the general academic vocabulary that lets them build arguments, report findings, hedge claims, and engage critically with published research. A student who knows “hypertension” but cannot use “significant,” “analysis,” “validity,” or “bias” in a sentence is missing the vocabulary infrastructure for scholarly medical communication.

Measuring academic vocabulary development requires a validated instrument. The Academic Word List (AWL), developed by Coxhead (8) through corpus analysis of 3.5 million words of academic text across four disciplines (Arts, Commerce, Law, and Science), is the most widely used framework for this purpose. In building the list, the 2,000 most frequent English words covered by the General Service List (9) were excluded. The AWL comprises 570 word families organized into 10 frequency-ranked sublists, where Sublist 1 contains the most frequent families and Sublist 10 the least frequent. Together, these 570 families account for roughly 10% of the running words in academic texts (10). The pedagogical logic is straightforward: a student whose writing reaches or approaches 10% AWL coverage is deploying the general academic vocabulary needed for scholarly discourse, while a student well below that threshold is not. The AWL has been validated across multiple disciplinary corpora: Chen and Ge (11) reported 10.07% coverage in medical research articles, Vongpumivitch et al. (12) found 11.17% in applied linguistics research papers, and Li and Qian (13) reported 10.46% in a financial services corpus, broadly consistent with the ∼10% benchmark, though with some disciplinary variation. Because some researchers considered the AWL too general for medical contexts, several field-specific lists were developed: the Medical Academic Word List of 623 word families covering 12.24% of medical research articles (14), a medical word list by Hsu (15), a medical academic vocabulary list (16), and a nursing vocabulary list by Yang (17). Hyland and Tse (18, 19) pushed back against the idea that the AWL works equally well across all fields, arguing that academic vocabulary is shaped by the discipline it appears in: a word like “significant” carries different meanings in different contexts. Wang and Nation (20) raised a related concern about homographs within the AWL. Nevertheless, the AWL remains the most widely used instrument for measuring academic vocabulary in learner writing, and it is the instrument adopted here. AWL coverage can be computed automatically and consistently from any collection of students’ writing. It applies consistently across academic disciplines and is well suited for longitudinal tracking across multiple instructional periods.

Research on AWL coverage in EFL health sciences students’ writing remains limited. Studies that track the same group of students across multiple instructional phases are even rarer. Pathan et al. (21) analyzed AWL coverage in doctoral theses written by non-native English speakers and found coverage rates consistently below expert benchmarks, indicating that even advanced L2 writers underuse academic vocabulary. Biber et al. (22) provided a useful longitudinal precedent by tracking grammatical complexity over two years in L2 English university students, though their focus was on grammar rather than vocabulary. Within health professions education, Wette (23) demonstrated that embedding academic literacy instruction into a first-year health sciences course led to measurable improvements in students’ ability to work with source texts. The scoping review by Klarare et al. (7) identified four main approaches for promoting academic literacies in health professions training: integrating literacy into existing courses, redesigning course structures, faculty–librarian collaboration, and offering dedicated literacy courses. The present study addresses this research gap by tracing vocabulary development in EFL health sciences students’ writing over four trimesters.

Layered on top of these challenges is the rapid arrival of AI writing tools. The release of ChatGPT in November 2022 introduced a new variable into EAP instruction. Large language models can produce polished academic English on demand, including the kind of vocabulary that EAP programs aim to develop over multiple semesters (24, 25). Kung et al. (25) demonstrated that ChatGPT could pass sections of the United States Medical Licensing Examination (USMLE), a finding that has since prompted extensive evaluation of large language models across diverse medical examination formats and question types (26). For academic vocabulary in particular, these tools create a fundamental tension that can be framed as two competing possibilities. Under the first, AI enhances learning: Shahsavar et al. (27) and Wang et al. (28) reported writing improvements when medical students used AI tools in structured settings, and sustained interaction with AI-generated academic text (reading, paraphrasing, editing, and critically engaging with it) could accelerate vocabulary acquisition under instructor guidance, consistent with usage-based accounts of language learning (29, 30).

Under the second possibility, AI substitutes for student effort. When a text reads well because a language model produced it, its vocabulary indexes the model's competence rather than the writer's. Apparent development is then an artefact of the tool, and the gap it conceals surfaces only when the student must write unaided: a case note, a discharge summary, an appraisal of a trial report. Morjaria et al. (31) found that AI-generated responses were at times indistinguishable from student-written ones in unsupervised assessments, and Doru et al. (32) found that even trained evaluators achieved inconsistent accuracy when attempting to detect AI-generated medical student essays. Nguyen (33) further cautioned that students who habitually accept AI output without independent verification may carry this pattern into clinical practice as automation bias. Boscardin et al. (24) nonetheless argued that AI literacy should be treated as a professional competency to be taught rather than a risk to be banned. Distinguishing genuine vocabulary acquisition from AI-assisted fluency therefore requires designs that compare student output across conditions of differing AI availability, which the setting of the present study is positioned to approximate.

Saudi Arabia provides a particularly informative setting for examining how AI availability interacts with vocabulary development. ChatGPT was not accessible in the Kingdom without a Virtual Private Network (VPN) when it first launched, and only became freely available in August 2023 (34). The KSAUHS Learner Corpus developed by Al Nafjan et al. (34), collected from September 2022 through December 2023, spans this entire transition. The same group of students produced writing during a period that moved from no AI access, through limited VPN-based access, to fully unrestricted availability. This amounts to a natural experiment that could not have been designed prospectively, since randomly assigning students to AI-available and AI-unavailable conditions would be neither practical nor ethical. This study takes advantage of this historical comparison to address three research questions: (i) what is the overall AWL coverage in EFL health sciences students’ writing, and how does it compare to expert benchmarks for medical academic texts?; (ii) how does AWL coverage develop across four trimesters, and does this development align with the phased introduction of ChatGPT in Saudi Arabia?; and (iii) which AWL word forms are most frequently used by health sciences students, and do they cluster into meaningful vocabulary domains? Besides answering these questions, the present study aims to provide health professions educators with evidence for curriculum design and vocabulary instruction priorities.

## Materials and methods

The data for this study come from the KSAUHS Learner Corpus (34), a longitudinal collection of EFL academic writing compiled at King Saud bin Abdulaziz University for Health Sciences (KSAUHS) in Saudi Arabia and managed in accordance with the FAIR data principles (35). The corpus was produced by 157 female preparatory program students, all with Arabic as their first language (34). The preparatory program is completed before students enter their health sciences specializations [Nursing, Applied Medical Sciences (AMS), Pharmacology, Dentistry, and Medicine]. The program is CEFR-aligned with an instructional range of approximately A2 to B2, spanning elementary to upper-intermediate proficiency. Corpus metadata additionally record each student's years of prior English schooling, other languages spoken, and preferred reference tools (34). Writing was collected electronically through the university's learning management system, and handwritten exam scripts were scanned and transcribed (34). All token counts reported here were derived from the author's own processing of the corpus texts; the four trimesters analysed in this study comprise 855,680 running words.

At the time of data collection, the preparatory program at KSAUHS ran on a trimester calendar across two academic years. The first year comprised three trimesters of English courses, and the second included a further writing course. The corpus spans these four consecutive trimesters, collected between Fall 2022 and Fall 2023. The first trimester focused on paragraph writing: students produced a practice paragraph, a general paragraph on topics such as being a good listener, and a cause-effect paragraph on topics related to student life (150–200 words each). The second trimester required a compare-contrast paragraph (e.g., gym vs. home workouts, a mother and father's roles) and a definition paragraph (e.g., mental health, an ideal roommate), followed by a timed three-hour exam in which students wrote two paragraphs by hand without access to language resources. The third trimester progressed to argumentative essay writing: two 450–500-word essays on topics related to education, digital learning, consumerism, and social image, plus an exam essay. The fourth trimester required students to select a published medical journal article and produce a summary, a critique with additional references, and a revised synthesis combining both. This progression from general paragraph writing to source-based medical academic writing is central to the study's design. Table 1 presents the corpus structure mapped against the four AI accessibility phases (34).

**Table 1:** Corpus design, writing tasks, and AI accessibility phases.

Trimester	Writing tasks	Words	AI accessibility phase	Key events
T1 (Sept–Dec 2022)	Practice paragraph; general paragraph; cause/effect paragraph (150–200 words each)	65,932	**Phase 1: Pre-AI**	ChatGPT released globally (Nov 2022); not accessible in Saudi Arabia without VPN
T2 (Dec 2022–Mar 2023)	Compare-contrast paragraph; definition paragraph; timed exam (two paragraphs)	204,951	**Phase 2: Early restricted**	ChatGPT exists but requires VPN
T3 (Mar–Jun 2023)	Argumentative essay 1 (education/digital learning); essay 2 (consumerism/social image); exam essay	342,380	**Phase 3: Increasing VPN access**	ChatGPT increasingly accessible via VPN; media coverage rising
T4 (Sept–Dec 2023)	Medical journal article summary; critique with additional references; revised synthesis	242,417	**Phase 4: Unrestricted**	ChatGPT fully accessible in Saudi Arabia (Aug 2023); no institutional AI restrictions on homework
Total	—	**855,680**	—	—

Bold indicates corpus totals.

AWL coverage was operationalized as the percentage of running words in the corpus that matched word forms from Coxhead's (8) 570 word families. Matching was case-insensitive and inclusive of both the American and British spellings. A minimum frequency threshold of 24 occurrences across the entire corpus was applied (equivalent to 2.80 per 100,000 words), which is proportionally consistent with the thresholds used by Coxhead (8) at 2.86 per 100,000 and Vongpumivitch et al. (12) at 3.33 per 100,000. Using Microsoft Excel, AWL coverage and frequency were computed both for the full corpus and for each trimester independently. The 100 most frequent AWL word forms were identified and organized by frequency, sublist membership, and peak trimester of occurrence. They were also grouped into thematic clusters based on semantic relatedness and disciplinary relevance to health sciences education.

## Results

The full corpus contained 855,680 words. After applying the frequency threshold, 65,574 AWL tokens were identified, resulting in an overall coverage of 7.66%. That puts KSAUHS students below the ∼10% benchmark that Coxhead and Byrd (10) reported for expert academic writing, and below the 10.07% that Chen and Ge (11) found in medical research articles. However, the AWL coverage in Table 2 shows a clear upward trajectory, rising from 3.69% in T1%–4.90% in T2, 7.45% in T3, and 11.39% in T4. The growth pattern is accelerating rather than linear: the increment between T1 and T2 was 1.21 percentage points, between T2 and T3 it was 2.55 percentage points, and between T3 and T4 it reached 3.94 percentage points. This convex growth curve suggests that each trimester produced a larger vocabulary gain than the one before it. By T4, student writing exceeded the 10.07% AWL benchmark that Chen and Ge (11) reported for published medical research articles. The differences in AWL coverage across the four trimesters were statistically significant (log-likelihood G^2^ = 8517.83, df = 3, *p* < 0.001), though the effect size was small (Cramér's V = 0.10), reflecting the fact that AWL items remain a minority of running words even as their share grows. The test shows the upward trend to be reliable, but it does not apportion the growth among its possible sources.

**Table 2:** AWL coverage by trimester with AI accessibility phase mapping.

Trimester	Total words	AWL tokens	AWL %	AI phase	Primary genre	*Δ* (%)
T1 (Fall 2022)	65,932	2,436	3.69%	Pre-AI	Short paragraphs	—
T2	204,951	10,034	4.90%	Early restricted	Paragraphs + exam	+1.21
T3	342,380	25,494	7.45%	Increasing VPN access	Essays + exam	+2.55
T4 (Fall 2023)	242,417	27,610	11.39%	Unrestricted	Journal summary/critique	+3.94
Full corpus	855,680	65,574	7.66%	—	—	—

The minimum occurrences of each word form in the entire corpus is 24.

### T1 (phase 1: pre-AI)

The most common words in T1 are everyday terms like good, people, time, and many. AWL words are scarce, which makes complete sense given that these students were at the very start of their EAP courses and the writing tasks were basic paragraphs on general topics. This trimester is a clean baseline because the first assignments were collected in September 2022, two months before ChatGPT was released anywhere in the world.

### T2 (phase 2: early restricted)

Coverage moves up slightly to 4.90%, mostly because of the shift to compare-contrast and definition paragraphs. These genres naturally pull in more AWL words. Health-related terms like “depression”, “mental”, “physical”, and “adults” start showing up here, reflecting essay topics on mental health and childhood. ChatGPT existed by this point but was blocked in Saudi Arabia unless students used a VPN. For most of them, that was enough of a barrier to keep AI influence negligible.

### T3 (phase 3: increasing VPN access)

The acceleration to 7.45% coincides with argumentative essay writing and growing ChatGPT accessibility. Like T4, T3 has its own exclusive vocabulary: 23 of the top 100 word forms appear only here, including “media” (810 tokens), “communication” (697), “academic” (605), “job” (438), “devices” (425), “negative” (413), and “issues” (413). These words reflect the essay topics (education, digital media, social image, consumerism) rather than medical reading. The genre shift to sustained argumentation is the most straightforward explanation for this jump, though some AI-mediated vocabulary enrichment in homework essays cannot be ruled out.

### T4 (phase 4: unrestricted AI)

The surge to 11.39% (+3.94 pp) coincides with both the most demanding genre (medical journal summary and critique) and fully unrestricted ChatGPT access. Remarkably, among the 73 unique word forms in the top 100 list (Supplementary Table 1), 25 appeared exclusively in T4 and did not occur elsewhere in the corpus. These are not marginal items. They include “participants” (1,299 tokens), “data” (1,018), “researchers” (599), “conducted” (444), “analysis” (371), “outcomes” (357), “bias” (264), “validity” (199), “method” (197), and “statistical” (186). This cluster represents the vocabulary of research methodology in journal articles that students were reading and summarizing for the first time.

The full-corpus sublist distribution reveals three rank-order inversions relative to the AWL's designed frequency hierarchy (Table 3). Sublist 4 (10.86%) outranks Sublist 3 (10.56%), driven by words such as “communication,” “stress,” “contrast,” and “summary” that the health sciences curriculum naturally elicits. Sublist 7 (7.99%) overtakes Sublist 6 (4.76%), largely because of “adults,” “media,” “intervention,” and “couples” that run through the T2–T4 writing topics. The most striking inversion involves Sublist 10, which outranks both Sublist 9 and Sublist 8. This is driven almost entirely by one word: “depression” accounts for 1,496 of Sublist 10's 1,680 tokens (89% of the total). For health sciences students, “depression” is not simply an academic vocabulary item; it is a core concept in their professional training, their essay topics, and the medical literature they encounter. These kinds of inversions have been reported in other AWL profiling studies (12) but take on particular significance here because they show how disciplinary context reshapes the vocabulary distribution that EAP programs need to address.

**Table 3:** AWL token distribution across ten sublists, ordered by corpus frequency.

AWL sublist	Total tokens	% of AWL tokens	Expected rank	Corpus rank	Inversion
1	17,016	25.96%	1	1	—
2	15,000	22.89%	2	2	—
4	7,117	10.86%	3	4	Yes (> Sub 3)
3	6,919	10.56%	4	3	Displaced
5	6,568	10.02%	5	5	—
7	5,240	7.99%	6	7	Yes (> Sub 6)
6	3,119	4.76%	7	6	Displaced
10	1,680	2.56%	8	10	Yes (> Sub 9 & 8)
9	1,648	2.51%	9	9	Yes (> Sub 8)
8	1,237	1.89%	10	8	Displaced

## Discussion

The results have several practical implications for health sciences education programs, EAP curriculum designers, and assessment committees. First, the data show that genre-progressive scaffolding works and should be standard practice. AWL coverage went from 3.69% to 11.39% over four trimesters, and the steepest gains came when students started working directly with published medical research. Wette (23) found similar results with source-based writing tasks in a first-year health sciences course, and Klarare et al.'s (7) scoping review identified embedding literacy into the curriculum as the most effective strategy. Programs that stop their EAP courses at the paragraph or essay stage and never ask students to engage with actual research articles are probably missing this opportunity to push vocabulary forward. On the basis of these data, at least one trimester of research-article work should come before clinical practice begins.

Second, vocabulary instruction should be health-domain specific. Two distinct AWL clusters emerged in the data. One is a health-psychology cluster (“mental,” “depression,” “physical,” “psychological,” “stress,” “medical”) that shows up naturally in T2 and T3 when students write about health-related topics. This cluster does not need much direct teaching because the curriculum already triggers it. The other is a research-methods cluster (“participants,” “data,” “analysis,” “significant,” “validity,” “bias”) that is heavily concentrated in T4. This one matters more for clinical readiness because it is the vocabulary students need for evidence-based practice (1, 2). Teaching these words explicitly before students encounter journal articles would make the transition smoother. The top 100 AWL list in Supplementary Table 1 provides a ready-made starting point for this kind of targeted instruction. Programs could also draw on the Medical Academic Word List (14) or Lei and Liu's (16) medical academic vocabulary list to supplement.

Third, AI-literate assessment is now a necessity rather than a future concern. The steepest increase in AWL coverage coincided with both the most demanding writing task and unrestricted ChatGPT access, which raises the question of how much of the Trimester 4 coverage reflects vocabulary the students have internalized and how much reflects the assistance of AI tools. Two readings are possible. Under the first, AI functions as a learning scaffold. Students who use ChatGPT to explore vocabulary, practise paraphrasing, and revise drafts under guidance may genuinely expand their productive repertoire. Shahsavar et al. (27) and Wang et al. (28) reported writing gains under such structured use. Under the second, AI enhances only the final product: a text may display sophisticated academic vocabulary because a language model supplied it, leaving the student's own competence unchanged and unmeasured. These possibilities are difficult to separate in unsupervised work. Morjaria et al. (31) showed that AI-generated responses were at times indistinguishable from student writing in unsupervised assessments, and Doru et al. (32) found that even experienced evaluators could not reliably detect the difference. For any program that relies on unsupervised writing to judge clinical readiness, this is a practical problem. Pairing unsupervised tasks with equivalent tasks completed under controlled conditions, where AI is unavailable, offers a workable check: a substantial drop in coverage would point to AI influence, whereas comparable results would suggest the gains are internalized. On balance, prohibiting AI is neither realistic nor clearly desirable; Boscardin et al. (24) argued that AI literacy belongs in the medical curriculum as a professional competency. The productive response is to teach with these tools while redesigning assessment so that it continues to measure what students can do unaided. Permitting AI is not the same as leaving it unregulated. Use can be allowed while remaining subject to disclosure of which tools were used and for what purpose, attribution of text that originated with a model, and assessment rules that state where AI is permitted and where it is not.

That overall coverage (7.66%) falls below the ∼10% expert benchmark (10, 11) is unsurprising for undergraduate EFL students still learning to write academic English while studying health sciences in a second language (3, 5). Interestingly, the four AI accessibility phases seem to line up with the four trimesters. T1 (pre-AI) provides a genuine baseline, all that writing was done before ChatGPT existed, or at least before anyone in Saudi Arabia could access it without technical expertise. The 3.69% coverage reflects what students could do on their own without AI assistance, when writing short paragraphs. T2 (early restricted) shows a modest increase (+1.21 pp) during a period when ChatGPT existed but needed a VPN to reach, for most students, that was a barrier high enough to keep AI influence negligible. T3 (increasing VPN access) is harder to read: the genre shifted to argumentative essays, VPN use was spreading, and both factors could have pushed coverage up. The genre explanation is simpler and probably accounts for most of the gain. T4 (unrestricted access) is where interpretation becomes hardest: the biggest increment (+3.94 pp), the most demanding genre, and full ChatGPT availability all arrive together.

Three factors could explain the Trimester 4 increase, and they cannot be separated with the present data. First, a genre effect: when students summarize and critique medical journal articles, the vocabulary the task requires (“participants,” “analysis,” “significant,” “bias”) is heavily AWL-indexed, so coverage rises almost as a property of the genre. Second, cumulative development: by Trimester 4 students had completed three trimesters of progressively more demanding EAP work, and a curve that steepens over time is what vocabulary-acquisition models predict (36). Third, AI availability: with ChatGPT freely accessible, some students may have used it for drafting, vocabulary consultation, or paraphrasing, which could raise coverage above what genre and development alone would produce. Because task complexity, cumulative instruction, and AI access all advanced over the same period, their contributions are confounded, and the design identifies an association rather than the effect of any single factor. Estimating their relative weight is the central question for follow-up work. One test is already possible within this corpus: comparing AWL coverage in Trimester 4 exam writing, produced under supervision without AI, against Trimester 4 homework, where AI use was possible.

The rank-order inversions are not merely a technical curiosity. The dominance of “depression” (Sublist 10) over all Sublist 8 and 9 words combined reflects what the curriculum foregrounded. Much of this prominence is topic-driven: several of the essay prompts, and the articles students were assigned to summarize and critique, concerned mental health, which would elicit this vocabulary directly. The frequency of “depression” therefore indexes the topics students were asked to write about at least as much as any independent lexical preference. The research-methods cluster (dominated by Sublist 1 and 2 words) carries clearer clinical relevance: “significant,” “validity,” and “bias” are the words that let practitioners distinguish sound evidence from weak. Taken together, these patterns support developing health-specific AWL supplementary materials that prioritize vocabulary of particular relevance to medical, nursing, and allied health training (14, 16, 17).

### Confidentiality and accountability in AI-assisted learning

The risks of AI-assisted learning in health sciences education are not only pedagogical. Anything entered into a commercial language model passes beyond the institution's control, and what students enter is not always harmless. A student who pastes a case description, a patient encounter, a classmate's draft, or details of a research participant into a public chatbot may release information that university policy and data protection law exist to safeguard. The tasks examined here dealt with general and academic subjects, so no such exposure occurred in this corpus. The question becomes live as soon as students begin writing clinical documentation, which follows within months of the program studied here. Teaching with these tools therefore carries responsibilities that teaching without them does not: deciding what may lawfully be entered into an external system, protecting patients, classmates, and research participants from disclosure, answering for the tools students are pointed toward, and setting rules for how AI-assisted work is declared. The vocabulary gains reported here are not by themselves a reason to adopt these tools.

### Limitations

Several limitations should be borne in mind. First, and most importantly, the study is observational, and the phased introduction of ChatGPT is confounded with two other variables that changed over the same period: the increasing complexity of the writing genres and the students’ cumulative language development. Because these three influences advanced together, the design cannot isolate the effect of AI availability, and the associations reported here should not be read as evidence that AI caused the observed vocabulary growth. Second, the most direct within-corpus check for AI-specific effects, comparing exam writing (produced without AI) against homework across trimesters, has not yet been carried out. Third, although the Academic Word List is well validated, it is a general instrument whose coverage is shaped by disciplinary context: curricular topics that happen to elicit AWL-listed items can raise coverage independently of a student's broader academic proficiency (18, 19). The prominence of mental-health vocabulary in this corpus is a concrete instance, since it is likely driven by the topics students were assigned rather than by an independent lexical choice. Fourth, the corpus-level significance test reported in the Results confirms that the upward trend is reliable, but it operates on aggregated token counts rather than on individual students; student-level modelling would be needed to estimate the effect while accounting for within-writer dependence. Together, these constraints temper the strength of the causal inferences that can be drawn and frame the results as associational.

### Future research directions

Several directions would strengthen and extend this work. The most immediate priority is a systematic exam-vs.-homework comparison of AWL coverage. Because students may have accessed AI tools before Trimester 4, this comparison should cover all four trimesters, and both homework and examination tasks, rather than Trimester 4 alone. A consistent gap between supervised and unsupervised writing would offer the clearest available evidence of AI-mediated inflation. It would also be valuable to disaggregate the corpus by health sciences specialization, to determine whether students entering nursing, dentistry, and medicine show different vocabulary trajectories. A further step would be to apply mixed-effects modelling that treats the individual student as the unit of analysis, complementing the corpus-level test reported here. Replication at other Saudi EMI institutions would help establish whether the patterns reported here are specific to this setting or generalize more broadly.

## Conclusion

This study offers the first longitudinal description of academic vocabulary development in EFL health sciences students’ writing across a full preparatory-year EAP sequence, and it does so during the period in which AI writing tools became widely available. Analyzing a corpus of 855,680 tokens compiled by Al Nafjan et al. (34), it found that AWL coverage rose from 3.69% to 11.39% across four trimesters, a statistically significant increase that, by the final trimester, surpassed published benchmarks for expert medical research articles. The study's contribution is twofold. Methodologically, it demonstrates that AWL coverage can track vocabulary growth sensitively across an entire EAP curriculum, and it provides a frequency-ranked list of the 100 most common AWL word forms in this population as a practical resource for curriculum designers. Substantively, it documents a trajectory shaped by three intertwined forces — progressively harder writing genres, cumulative language development, and the phased arrival of ChatGPT — whose individual contributions the present design cannot separate. This inseparability is both the study's principal limitation and the reason its setting is unusual, since few datasets capture the same cohort writing under conditions that shifted from no AI access to unrestricted access. For health professions educators, three implications follow. First, EAP curricula should be sequenced deliberately toward research-literacy genres, since the sharpest vocabulary gains accompanied engagement with authentic medical articles. Second, the vocabulary most critical for evidence-based practice — research-methods terms such as “participants,” “significant,” “validity,” and “bias” — should be taught explicitly rather than left to incidental acquisition. Third, and most urgently, writing assessments should be redesigned so that vocabulary displayed under unsupervised conditions is corroborated by performance under supervision, since apparent competence may increasingly reflect AI assistance. Future work that separates these factors, through supervised-vs.-unsupervised comparisons and student-level statistical modelling, will determine how much of the growth documented here represents durable acquisition. Establishing that distinction will matter as much to the integrity of clinical assessment as it does to the teaching of academic English.

## Data Availability

The data analysed in this study are drawn from the KSAUHS Learner Corpus (34). Inquiries regarding access to the corpus should be directed to the original corpus developers.
