# Supplementary material for: From writing paragraphs to evaluating articles: academic vocabulary development in EFL health sciences students during the ChatGPT transition
Source: Front Med (Lausanne). 2026 Sep 16;13:1895483. doi: 10.3389/fmed.2026.1895483 (PMC13623714; doi:10.3389/fmed.2026.1895483)
Supplement: Supplementary file 1 [file Table1.docx]

**Supplementary material**

*Supplementary Table S1*  The 100 most frequent AWL word forms in the KSAUHS Learner Corpus, with frequencies, sublists, and peak trimester of occurrence.

| **#** | **Word form** | **Freq.** | **Sublist** | **Peak T** | **Thematic cluster** |
| --- | --- | --- | --- | --- | --- |
| 1 | participants | 1,299 | Sub 2 | T4 | Research methods |
| 2 | mental | 1,101 | Sub 5 | T3 | Health-psychology |
| 3 | research | 1,032 | Sub 1 | T4 | Research methods |
| 4 | data | 1,018 | Sub 1 | T4 | Research methods |
| 5 | media | 810 | Sub 7 | T3 | Social-digital |
| 6 | depression | 729 | Sub 10 | T4 | Health-psychology |
| 7 | communication | 697 | Sub 4 | T3 | Social-digital |
| 8 | academic | 605 | Sub 5 | T3 | Academic discourse |
| 9 | researchers | 599 | Sub 1 | T4 | Research methods |
| 10 | affect | 566 | Sub 2 | T3 | Cause-effect |
| 11 | physical | 513 | Sub 3 | T3 | Health-psychology |
| 12 | physical | 496 | Sub 3 | T4 | Health-psychology |
| 13 | depression | 486 | Sub 10 | T2 | Health-psychology |
| 14 | mental | 469 | Sub 5 | T2 | Health-psychology |
| 15 | significant | 465 | Sub 1 | T4 | Research methods |
| 16 | finally | 455 | Sub 2 | T2 | Academic discourse |
| 17 | mental | 454 | Sub 5 | T4 | Health-psychology |
| 18 | affects | 451 | Sub 2 | T3 | Cause-effect |
| 19 | conducted | 444 | Sub 2 | T4 | Research methods |
| 20 | job | 438 | Sub 4 | T3 | Social-digital |
| 21 | devices | 425 | Sub 9 | T3 | Social-digital |
| 22 | negative | 413 | Sub 3 | T3 | Cause-effect |
| 23 | issues | 413 | Sub 1 | T3 | Academic discourse |
| 24 | individuals | 403 | Sub 1 | T3 | Academic discourse |
| 25 | conclusion | 394 | Sub 2 | T3 | Academic discourse |
| 26 | factors | 394 | Sub 1 | T4 | Research methods |
| 27 | impact | 387 | Sub 2 | T4 | Cause-effect |
| 28 | consequences | 385 | Sub 2 | T3 | Cause-effect |
| 29 | adults | 380 | Sub 7 | T3 | Health-psychology |
| 30 | communicate | 378 | Sub 4 | T3 | Social-digital |
| 31 | factors | 375 | Sub 1 | T3 | Research methods |
| 32 | analysis | 371 | Sub 1 | T4 | Research methods |
| 33 | benefits | 358 | Sub 1 | T3 | Cause-effect |
| 34 | outcomes | 357 | Sub 3 | T4 | Research methods |
| 35 | grades | 355 | Sub 7 | T3 | Social-digital |
| 36 | psychological | 352 | Sub 5 | T3 | Health-psychology |
| 37 | instance | 343 | Sub 3 | T2 | Academic discourse |
| 38 | impact | 343 | Sub 2 | T3 | Cause-effect |
| 39 | role | 333 | Sub 1 | T2 | Academic discourse |
| 40 | couples | 331 | Sub 7 | T3 | Health-psychology |
| 41 | intervention | 329 | Sub 7 | T4 | Research methods |
| 42 | instance | 322 | Sub 3 | T3 | Academic discourse |
| 43 | design | 322 | Sub 2 | T4 | Research methods |
| 44 | financial | 318 | Sub 1 | T3 | Social-digital |
| 45 | adulthood | 316 | Sub 7 | T2 | Health-psychology |
| 46 | significant | 314 | Sub 1 | T3 | Research methods |
| 47 | found | 312 | Sub 9 | T4 | Research methods |
| 48 | stress | 298 | Sub 4 | T3 | Health-psychology |
| 49 | medical | 289 | Sub 5 | T3 | Health-psychology |
| 50 | physical | 287 | Sub 3 | T2 | Health-psychology |
| 51 | stress | 286 | Sub 4 | T4 | Health-psychology |
| 52 | cultures | 282 | Sub 2 | T3 | Social-digital |
| 53 | depression | 281 | Sub 10 | T3 | Health-psychology |
| 54 | major | 278 | Sub 1 | T3 | Academic discourse |
| 55 | benefits | 269 | Sub 1 | T2 | Cause-effect |
| 56 | medical | 268 | Sub 5 | T4 | Health-psychology |
| 57 | bias | 264 | Sub 8 | T4 | Research methods |
| 58 | contrast | 263 | Sub 4 | T2 | Academic discourse |
| 59 | previous | 260 | Sub 2 | T4 | Research methods |
| 60 | volunteering | 256 | Sub 7 | T2 | Social-digital |
| 61 | individuals | 252 | Sub 1 | T4 | Academic discourse |
| 62 | furthermore | 245 | Sub 6 | T3 | Academic discourse |
| 63 | conclusion | 240 | Sub 2 | T4 | Academic discourse |
| 64 | environment | 238 | Sub 1 | T3 | Social-digital |
| 65 | duration | 238 | Sub 9 | T4 | Research methods |
| 66 | benefits | 234 | Sub 1 | T1 | Cause-effect |
| 67 | negatively | 232 | Sub 3 | T3 | Cause-effect |
| 68 | sum | 230 | Sub 4 | T2 | Academic discourse |
| 69 | finally | 229 | Sub 2 | T1 | Academic discourse |
| 70 | illustrate | 229 | Sub 3 | T3 | Academic discourse |
| 71 | adults | 217 | Sub 7 | T2 | Health-psychology |
| 72 | authors | 215 | Sub 6 | T4 | Research methods |
| 73 | final | 214 | Sub 2 | T4 | Academic discourse |
| 74 | potential | 213 | Sub 2 | T4 | Research methods |
| 75 | affect | 211 | Sub 2 | T4 | Cause-effect |
| 76 | research | 208 | Sub 1 | T3 | Research methods |
| 77 | specific | 206 | Sub 1 | T4 | Research methods |
| 78 | psychological | 206 | Sub 5 | T4 | Health-psychology |
| 79 | project | 201 | Sub 4 | T4 | Research methods |
| 80 | validity | 199 | Sub 3 | T4 | Research methods |
| 81 | technology | 197 | Sub 3 | T3 | Social-digital |
| 82 | accurate | 197 | Sub 6 | T4 | Research methods |
| 83 | method | 197 | Sub 1 | T4 | Research methods |
| 84 | instance | 196 | Sub 3 | T1 | Academic discourse |
| 85 | furthermore | 190 | Sub 6 | T4 | Academic discourse |
| 86 | conclusion | 188 | Sub 2 | T2 | Academic discourse |
| 87 | culture | 188 | Sub 2 | T3 | Social-digital |
| 88 | statistical | 186 | Sub 4 | T4 | Research methods |
| 89 | period | 181 | Sub 1 | T4 | Research methods |
| 90 | gender | 180 | Sub 6 | T4 | Research methods |
| 91 | affected | 178 | Sub 2 | T3 | Cause-effect |
| 92 | interventions | 175 | Sub 7 | T4 | Research methods |
| 93 | summary | 173 | Sub 4 | T4 | Research methods |
| 94 | jobs | 172 | Sub 4 | T3 | Social-digital |
| 95 | transportation | 170 | Sub 6 | T3 | Social-digital |
| 96 | individual | 169 | Sub 1 | T3 | Academic discourse |
| 97 | adults | 169 | Sub 7 | T4 | Health-psychology |
| 98 | affected | 169 | Sub 2 | T4 | Cause-effect |
| 99 | focus | 166 | Sub 2 | T3 | Academic discourse |
| 100 | jobs | 156 | Sub 4 | T2 | Social-digital |
